# Contribution of the classical polymerase chain reaction in the diagnosis of a HIV-1 infected patient in Benin: a case report

**DOI:** 10.1186/s12981-021-00342-2

**Published:** 2021-04-21

**Authors:** Edmond Tchiakpe, René K. Keke, Nicole Vidal, Moussa Bachabi, Flore Armande Gangbo, Halimatou Diop‑Ndiaye, Coumba Toure‑Kane, Akadiri Yessoufou

**Affiliations:** 1 Laboratory of Cell Biology and Physiology, Department of Biochemistry and Cellular Biology, Faculty of Sciences and Technology (FAST), Institute of Applied Biomedical Sciences (ISBA), University of Abomey-Calavi, Calavi, 01 BP 918 Cotonou, Benin; 2 National Reference Laboratory of Health Program Fighting Against AIDS in Benin (LNR/PSLS), Health Ministry of Benin, BP 1258 Cotonou, Benin; 3 UMI233-TransVIHMI, IRD (Institut de Recherche Pour Le Développement), U1175 (INSERM) et, Université de Montpellier, Montpellier, France; 4 Health Program Fighting Against AIDS in Benin (PSLS), Health Ministry of Benin, Cotonou, Benin; 5 Institute for Health Research, Epidemiological Surveillance and Training of Senegal, Dakar, Senegal

**Keywords:** HIV-1, Serology inconclusive, Viral load, DNA sequencing, Benin

## Abstract

**Background:**

First ambitious target by 2020 of UNAIDS is that 90% of people living with HIV know their HIV status. In people older than 18 months of age, serological confirmation test is recommended to confirm HIV infection*.*

**Case presentation:**

Here we report the case of a patient tested positive with HIV-1, ELISA, Murex^®^ Ag⁄Ab Combination assay (OD450 = 0.802 and cutoff-OD = 0.279) and negative by using FIRST RESPONSE HIV1-2.O CARD TEST (version 2.0) RAPID HIV CARD TEST. Viral load performed with Cobas^®^ TaqMan^®^ 96/Cobas^®^ Ampliprep^®^ was 6.49log_10._ The virus could be sequenced in partial *gag* and *pol* genes and belonged to CRF02_AG clade.

**Conclusion:**

Conventional PCR is a complementary method for the diagnosis of inconclusive HIV-1 serologies by antibodies.

## Background

The first ambitious target by 2020 of UNAIDS is that 90% of people living with HIV know their HIV status (https://www.unaids.org/fr/resources/documents/2014/90-90-90). In people older than 18 months of age, serological confirmation test is recommended to confirm HIV infection. However, the use of DNA and RNA PCR is essential in case of low level of antibodies during the window period or at the final stage of HIV infection or in the acute phase of infection [1]**.** Here, we report the case of a patient with indeterminate results in antibodies HIV-1 serology but positive for classical RNA PCR.

## Case presentation

To our Knowledge, the HIV screening algorithm in Benin uses a sensitivity test including the VIKIA and a specificity test including the First response in the peripheral laboratories of Benin. If the VIKIA test is negative, a negative result is reported. If Vikia is positive, the specimen is tested at First response. If First response is positive, a positive result is reported. If First response is negative (discordant), an indeterminate result is reported. The specimen is retested two and four months later. If the results remain unchanged, the specimen is sent to the LNR for testing with ELISA and WB. Molecular testing is used if the result remains the same as that found in peripheral laboratories.

A 25-year old man was referred to HIV confirmation test in National Laboratory Fighting Against AIDS (LNR/PSLS) in Benin for HIV detection because his HIV infection diagnosis was indeterminate in remote area laboratory, albeit he had no clinical symptoms. But he declared had unprotected intercourse with female sex workers.

The sample revealed inconsistent results for serological testing. In fact, the HIV-1/2 antibody was positive by enzyme-linked immunosorbent assay (ELISA, Murex^®^ Ag⁄Ab Combination assay, DiaSorin S.p.A. Via Crescentino 13040 Saluggia (VC)—Italy) and negative in (FIRST RESPONSE HIV1-2.O CARD TEST (version 2.0) RAPID HIV CARD TEST., Valsad, Gujarat, India). Werstern Blot (INNO-LIA^™*^HIV I/II Score) (WB) result analysis was indeterminate with P24 ( ±).

Two and four months later, news samples were taken and recorded under two different numbers based on the patient registration system in the LNR/PSLS (No. 6652 and No. 8285 respectively) and re-analyzed. The ELISA were still positive (OD450 = 0.802 and cutoff-OD = 0.279) and the FIRST RESPONSE were negative. WB were still indeterminate P24 ( ±).

Plasma HIV-1 RNA viral load (VL) were performed using Cobas^®^ TaqMan^®^ 96/Cobas^®^ Ampliprep^®^ (CAP/CAP-CTM) HIV-1 quantitative assay (Roche Molecular Diagnostics, Basel, Switzerland) in LNR/PSLS and gave 6.49log_10_. The linear range of 20–10.000.000 cp/ml with a detection limit of 20 cp/ml was defined by the manufacturers.

**Fig. 1:**
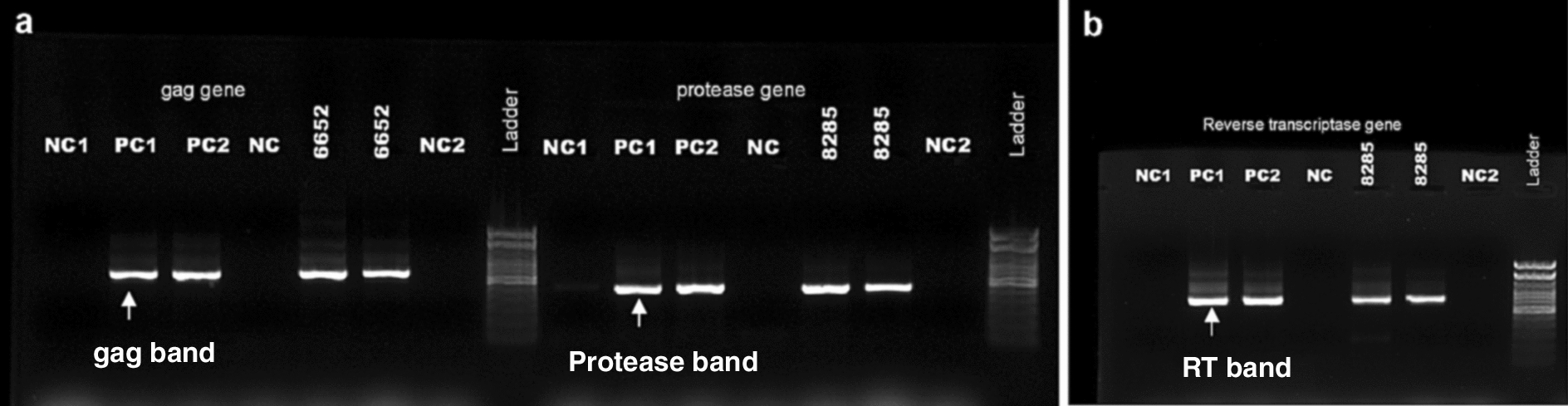
Agarose gel showing migration of amplified genes. **a** Gag and protease genes. **b** Reverse transcriptase gene *NC1* mix 1 negative control, *NC2* mix 2 negative control, *PC1* positive control 1, *PC2* positive control 2, *NC* negative control extracted at the same time as the samples, *RT* reverse transcriptase

Based on the contradictory results between serological and virological tests, HIV-RNA were extracted from plasma by using the QIAmp Viral RNA kit (Qiagen, Courtaboeuf, France) according to the manufacturer’s instructions in LNR/PSLS. Nested PCR was performed in LNR/PSLS on the *gag* gene (sample No. 6652) (Fig. 1a), the entire protease (sample N°8285) (Fig. 1b) and the first 240 amino acids encoding the reverse transcriptase (RT) (sample No. 8285) with primers as previously described [2] (Fig. 1b)**.**

PCR products were purified (Qiagen) and sequenced on AB 3500 Genetic Analyzer using Big Dye Terminator v3.1 (Applied Biosystems, Courtaboeuf, France). Sequences were edited online (https://pssm.cfenet.ubc.ca/account/login) and translated into amino acids.

No major drug resistance mutation was observed in protease and RT genes. Phylogenetic analysis showed phylogenetic clustering with CRF02_AG clade in both *gag* and partial *pol* genes (GenBank accession no. MT594403, MT800817).

## Discussion and conclusions

This work presents the case of a man repeatedly positive in ELISA HIV, negative with first response and indeterminate at WB HIV with plasma HIV-1 viral load by CAP/CAP-CTM of 6.49log_10_. Sequencing *gag* and partial *pol* genes showed CRF02_AG.

The HIV diagnostic infection tests are regularly improved to identify all cases of infection. Despite the use of 4th generation ELISA tests, cases of indeterminate serology continue to exist and constitute a challenge for many national AIDS programs to reach the first 90 of the UNAIDS 3X90 target. This can be explained by the low level of antibody expression during the window period or terminal stage of HIV infection or acute HIV infection [3]. The use of plasma HIV-1 RNA viral load quantification and classical PCR become essential tools to diagnose these cases of inconclusive serology. The FIRST RESPONSE showed negative result in our case report. The first response was evaluated in the study conducted by Iqbal et al., and showed its ability to detect all positive and negative cases of HIV with a sensitivity and specificity of 100% [4]. Indeed, the high sensitivity is recommended in screening tests. In this same study, the high sensitivity of FIRST RESPONSE was to be able to identify an HIV-1-indeterminate specimen because its principle of immunochromatography works with gp41, p24 including subtype O, gp36 antigens different to gp41, gp120, and gp36 in HIV TRI-DOT [4]. 

Our case VL was 6.49log_10_. This may suggest that the patient was at an early stage of infection. Indeed, high VL is associated with an early stage of infection in patients with inconclusive serology [5]. HIV-1 genetic diversity is a significant concern with respect to diagnostic, and we believe that CAP/CTM v2.0 using two double-labeling hybridization probes and targeting both the *gag* and LTR regions, amplified and detected correctly our study’s strain. However, cases of underestimation of viral load results for non-clade B samples have been described [6]. Pierce et al. reported in Philadelphia three cases classified as indeterminate in WB but VL were detected with Gen-Probe Aptima HIV-1 RNA qualitative assay [7]. The use of VL and DNA sequencing is necessary in the context of increasing inconclusive HIV testing serologies. That is why Aptima HIV assay is used in situations where HIV-1 antibodies were not present during the diagnosis of acute or primary infection in symptomatic patients [8]**.**

In our case, both gag and partial pol HIV-1 genes have been amplified and due to the success of the nucleotide sequencing by the Sanger dideoxy technical and the CRF02_AG subtype identified on both genes, we concluded that the virus present in our patient is a variants with prevalence > 20% [9, 10].

Since the diagnosis was made, the patient has benefited from ART treatment in health facilitie care in Cotonou until today.

In conclusion, our patient is an unusual case demonstrating the use of additional tests such as classical HIV-1 DNA or RNA PCRs to diagnose HIV-1 infection in order to reduce the number of HIV-1 indeterminate results.

## Data Availability

All the raw data generated are available upon reasonable request to corresponding Author.

## Abbreviations

ELISA: Enzyme-linked immunosorbent assay
UNAIDS: United Nations Programme on HIV/AIDS
RNA: Ribonucleic acid
PCR: Polymerase chain reaction
HIV: Human immunodeficiency viruse
LNR: National reference laboratory
PSLS: Health program fighting against AIDS in Benin
P24: Protein 24
VL: Viral load
RT: Reverse transcriptase
AIDS: Acquired immune deficiency syndrome
LTR: Long terminal repeat
DNA: Dexoyribonucleic acids
OD: Optical density

## References

[CR1] Shen L, Liu X, Wang T, Yang J, Xiao W, Mao L (2018). tradictory results of serological confirmatory test and real-time PCR assays in diagnosis a patient of HIV-1. Int J Infect Dis.

[2] Bakhouch K, Oulad-Lahcen A, Bensghir R, Blaghen M, Elfilali KM, Ezzikouri S (2009). The prevalence of resistance-associated mutations to protease and reverse transcriptase inhibitors in treatment-naive (HIV1)-infected individuals in Casablanca, Morocco. J Infect Dev Ctries.

[3] Mellors JW, Rinaldo CR, Gupta P, White RM, Todd JA, Kingsley LA (1996). Prognosis in HIV-1 infection predicted by the quantity of virus in plasma. Science.

[4] Syed Iqbal H, Balakrishnan P, Murugavel KG, Suniti S (2008). Performance Characteristics of a new Rapid Immunochromatographic Test for the detection of Antibodies to Human Immunodeficiency Virus (HIV) Types 1 and 2. J Clin Lab Anal..

[5] Selik RM, Linley L (2018). Viral loads within 6 weeks after diagnosis of HIV infection in early and later stages: observational study using national surveillance data. JMIR Public Health Surveill..

[6] Church D, Gregson D, Lloyd T, Klein M, Beckthold B, Laupland K, Gill MJ (2011). Comparison of the RealTime HIV-1, COBAS TaqMan 48 v1.0, Easy Q v1.2, and Versant v3.0 assays for determination of HIV-1 viral loads in a cohort of Canadian patients with diverse HIV subtype infections. J Clin Microbiol..

[7] Pierce VM, Neide B, Hodinka RL (2011). Evaluation of the Gen-Probe Aptima HIV-1 RNA qualitative assay as an alternative to Western blot analysis for confirmation of HIV infection. J Clin Microbiol..

[8] CDC (1987). Perspectives in disease prevention and health promotion public health service guidelines for counseling and antibody testing to prevent HIV infection and AIDS. MMWR Morb Mortal Wkly Rep..

[9] Larder BA, Kohli A, Kellam P, Kemp SD, Kronick M, Henfrey RD (1993). Quantitative detection of HIV-1 drug resistance mutations by automated DNA sequencing. Nature.

[10] Palmer S, Kearney M, Maldarelli F, Halvas EK, Bixby CJ, Bazmi H (2005). Multiple, linked human immunodeficiency virus type 1 drug resistance mutations in treatment-experienced patients are missed by standard genotype analysis. J Clin Microbiol.

